# Genetically-Engineered Pig-to-Baboon Liver Xenotransplantation: Histopathology of Xenografts and Native Organs

**DOI:** 10.1371/journal.pone.0029720

**Published:** 2012-01-11

**Authors:** Burcin Ekser, Edwin Klein, Jing He, Donna B. Stolz, Gabriel J. Echeverri, Cassandra Long, Chih Che Lin, Mohamed Ezzelarab, Hidetaka Hara, Massimiliano Veroux, David Ayares, David K. C. Cooper, Bruno Gridelli

**Affiliations:** 1 Thomas E. Starzl Transplantation Institute, University of Pittsburgh Medical Center, Pittsburgh, Pennsylvania, United States of America; 2 Department of Surgery, Transplantation and Advanced Technologies, Vascular Surgery and Organ Transplant Unit, University Hospital of Catania, Catania, Italy; 3 Division of Laboratory Animal Resources, University of Pittsburgh, Pittsburgh, Pennsylvania, United States of America; 4 Division of Immunogenetics, Department of Pediatrics, Children's Hospital of Pittsburgh, Pittsburgh, Pennsylvania, United States of America; 5 Department of Cell Biology and Physiology, University of Pittsburgh, Pittsburgh, Pennsylvania, United States of America; 6 Revivicor Inc., Blacksburg, Virginia, United States of America; 7 Mediterranean Institute for Transplantation and Advanced Specialized Therapies (ISMETT), Palermo, Italy; University of Colorado School of Medicine, United States of America

## Abstract

Orthotopic liver transplantation was carried out in baboons using wild-type (WT, n = 1) or genetically-engineered pigs (α1,3-galactosyltransferase gene-knockout, GTKO), n = 1; GTKO pigs transgenic for human CD46, n = 7) and a clinically-acceptable immunosuppressive regimen. Biopsies were obtained from the WT pig liver pre-Tx and at 30 min, 1, 2, 3, 4 and 5 h post-transplantation. Biopsies of genetically-engineered livers were obtained pre-Tx, 2 h after reperfusion and at necropsy (4–7 days after transplantation). Tissues were examined by light, confocal, and electron microscopy. All major native organs were also examined. The WT pig liver underwent hyperacute rejection. After genetically-engineered pig liver transplantation, hyperacute rejection did not occur. Survival was limited to 4–7 days due to repeated spontaneous bleeding in the liver and native organs (as a result of profound thrombocytopenia) which necessitated euthanasia. At 2 h, graft histology was largely normal. At necropsy, genetically-engineered pig livers showed hemorrhagic necrosis, platelet aggregation, platelet-fibrin thrombi, monocyte/macrophage margination mainly in liver sinusoids, and vascular endothelial cell hypertrophy, confirmed by confocal and electron microscopy. Immunohistochemistry showed minimal deposition of IgM, and almost absence of IgG, C3, C4d, C5b-9, and of a cellular infiltrate, suggesting that neither antibody- nor cell-mediated rejection played a major role.

## Introduction

The ultimate therapy for end-stage liver disease is orthotopic liver allotransplantation, but this therapy is limited by an inadequate number of livers from deceased donors. During the past 13 years, approximately 30,000 patients died while waiting for a suitable donor liver (www.unos.org). Patients with acute liver failure may need to undergo transplantation (Tx) urgently, sometimes within 24–36 h [1]. Xenotransplantation (xenoTx) would clearly solve the problem of donor supply as organs would be available whenever needed. As an initial clinical trial, pig livers could be used for patients with acute hepatic failure as a “bridge” to alloTx [1].

The Tx of kidneys and hearts from α1,3-galactosyltransferase gene-knockout (GTKO) pigs [2] and pigs expressing a human complement-regulatory protein [3], [4] into nonhuman primates (NHPs) has largely overcome hyperacute rejection (HAR) [5]–[9]. There is very little experience of pig-to-NHP liver Tx [10]–[11]. The use of genetically-engineered (GE) pigs for liver Tx, where the donor pig expressed human CD55 [12] or the combination of CD55, CD59, and H-transferase [13], has been reported by one group, and we have recently reported the results of Tx of GTKO and GTKO/CD46 pig livers in baboons [14]. Survival has not extended beyond 8 days. In our own studies, survival of recipient baboons (4–7 days) was limited by spontaneous bleeding occurring in body cavities, native organs, and the graft as a consequence of a profound thrombocytopenia, which developed within 1 h after reperfusion [14].

There have been a few descriptions of the histopathology of wild-type (WT) pig-to-NHP liver xenoTx, mainly describing HAR [12], [15]–[19], but almost no information on the histopathology of GE pig livers following xenoTx. We here describe the histopathology of (i) a WT pig liver after xenoTx, with serial biopsies to evaluate the development of HAR, and (ii) GTKO or GTKO/CD46 pig livers following Tx into baboons. We also report the histopathological features in the major native organs of the baboon recipients. Although some histopathological features were briefly mentioned in our previous publication [14], we now provide a full report.

## Materials and Methods

### Animals

Baboons (*Papio* species) (Oklahoma University Health Sciences Center, Oklahoma City, OK) of either sex, weighing 7–12 kg, aged 2.70–3.47 years were recipients of one allograft and 9 xenografts, and donors of one liver (Table 1). Wild-type (genetically-unmodified, WT) Landrace/large white pigs (Country View Farm, Schellsburg, PA) were donors of liver allografts (n = 2) and a xenograft (n = 1), and recipients of 2 allografts. GTKO (n = 1) or GTKO/CD46 (n = 7) pigs (Revivicor, Blacksburg, VA) were sources of xenograft livers (Table 1) [14]. From our original study [14], two liver xenotransplants have been excluded from the present report as donor-recipient size-mismatch precluded abdominal closure, necessitating early termination of the experiment.

**Table 1:** Recipient and donor information, immunosuppressive regimen and biopsy time-points.

Recipient #	Donor #	Donor Species Type	Immunosuppressive Therapy	Survival (days)	Liver Biopsy Time-points
**Allotransplantation**					
P40307	P40107	WT	None	>3	Pre-Tx, 2 h, euthanasia
P40207	P40407	WT	None	>2	Pre-Tx, 2 h, euthanasia
B3408	B16407	Baboon	ATG+TAC+MMF+CS	>31	Pre-Tx, 2 h, day 22, euthanasia
**Xenotransplantation**					
B16907	P3008	WT	None	5 h	Pre-Tx, 30 min, 1 h, 2 h, 3 h, 4 h, 5 h
B3108	P8208	GTKO	ATG+TAC+MMF+CS	6	Pre-Tx, 2 h, euthanasia#
B3208	P14508	GTKO/CD46	ATG+TAC+MMF+CS	4	Pre-Tx, 2 h, euthanasia#
B7708	P21708	GTKO/CD46	ATG+TAC+MMF+CS	7	Pre-Tx, 2 h, euthanasia#
B7808	P22108	GTKO/CD46	ATG+TAC+MMF+CS	6	Pre-Tx, 2 h, euthanasia#
B7908	P26708	GTKO/CD46	CyP+TAC+MMF+CS+(CL for donor)	<1	Pre-Tx, 2 h, euthanasia
B8108	P26608	GTKO/CD46	CyP+TAC+MMF+CS+(CL for donor)	1	Pre-Tx, 2 h, euthanasia
B18508	P3909	GTKO/CD46	ATG+TAC+MMF+CS+CVF	5	Pre-Tx, 2 h, euthanasia#
B18908	P4009	GTKO/CD46	CyP+TAC+MMF+CS	6	Pre-Tx, 2 h, euthanasia#

P = pig, B = baboon, ATG = antithymocyte globulin, TAC = tacrolimus, MMF = mycophenolate mofetil, CS = corticosteroids, CVF = cobra venom factor, CyP = cyclophosphamide, CL = clodronate liposomes, Tx = transplantation, # = native organs were biopsied at necropsy (euthanasia).

All animal care was in accordance with the Principles of Laboratory Animal Care formulated by the National Society for Medical Research and the Guide for the Care and Use of Laboratory Animals prepared by the Institute of Laboratory Animal Resources and published by the National Institutes of Health (NIH publication No. 86–23, revised 1985). Protocols were approved by the University of Pittsburgh Institutional Animal Care and Use Committee (IACUC# 0706493). The animal experiments were conducted in strict compliance with animal welfare regulations, and the steps taken to ameliorate suffering were in accordance with the recommendations of the Weatherall Report on the use of non-human primates in research [30] as well as with the ARRIVE guidelines on animal research [31]. All recipient baboons placed in a tether-jacket system, as previously described [5]–[7], and so they did not require to be sedated for each blood draws throughout the experiment. Biopsy samples were taken under anesthesia or at the time of necropsy (see below) avoiding any suffering to the animal. All animals were given Buprenorphine 0.01 mg/kg i.m. twice daily for 3 days after any surgical intervention for analgesia.

### Orthotopic liver transplantation

Two pig allotransplants and one WT pig-to-baboon liver xenoTx were performed without immunosuppression (Table 1). Nine immunosuppressed baboons received orthotopic liver grafts from a baboon (n = 1) or GTKO pig (n = 1) or GTKO/CD46 pigs (n = 7). All transplants were carried out using the standard surgical technique of orthotopic liver Tx after recipient hepatectomy [14]. All xenograft recipients except one (B3108) underwent splenectomy immediately after reperfusion of the liver. Details of the clinically-applicable immunosuppressive protocols are shown in Table 1.

### Timing of biopsies of liver grafts and major native organs

Table 1 summarizes the timing of liver biopsies. All biopsies (wedge) were obtained under direct vision, except in the baboon allograft (B3408) on post-operative day 22, which was a transcutaneous Tru-cut needle biopsy. The WT pig liver underwent serial biopsies at 30 min, 1, 2, 3, 4 and 5 h. Biopsies of GTKO and GTKO/CD46 pig livers were obtained pre-Tx, approximately 2 h after reperfusion (just before abdominal closure), and at necropsy. At necropsy, the macroscopic appearance of the liver graft was differentiated into (i) *light* areas, indicating relatively normal appearance, and (ii) *dark* areas that proved to be areas of hemorrhage. All major native organs were examined, and biopsies were taken from the areas that appeared most affected by pathogenic processes, e.g., hemorrhage.

### Light microscopy

Liver tissues were stored in 10% formalin for subsequent light microscopy. Paraffin blocks were prepared, 4 µm sections cut and stained with hematoxylin and eosin (H&E). On examination, particular attention was paid to (i) the presence of cellular infiltrates (neutrophils, lymphocytes, eosinophils), (ii) blood vessels (congestion, hemorrhage, fibrin aggregation, venulitis or arteritis), (iii) hepatocytes (vacuolation, necrosis), (iv) bile ducts (inflammation, necrosis), and (v) interstitial tissue (fibrosis). All major recipient organs were examined. Staining for iron deposition was carried out in some cases.

### Immunohistopathology

For immunohistochemistry studies, liver biopsies were stored at −80°C until processed. Cryosections (8 µm) were cut and mounted on to gelatin-coated slides. After being fixed in 2% paraformaldehyde in PBS for 15 min, sections were blocked with 20% non-immune normal donkey serum for 1 h at room temperature. Primary monoclonal antibodies were used to demonstrate (i) a cellular response (T cells [CD3], B cells [CD20], macrophages [CD68], neutrophils [CD97]), (ii) antibody and complement deposition (IgM, IgG, IgE, C3, C4d, C5b-9), and (iii) the presence of thrombotic microangiopathy (aggregation of platelets [CD41] and fibrin deposition) (Table 2), as previously described [7], [8], [19]. After washing in PBS, sections were incubated with different secondary antibodies. Table 3 summarizes the primary and secondary antibodies used, with their concentrations and sources.

**Table 2:** Liver immunohistopathology at 2 h and at necropsy.

				Cellular Response					Antibody deposition			Complement deposition			Thrombotic microangiopathy	
Donor Species Type	Pig#	Baboon#	Time of Bx	CD3	CD20	CD68 (pig)	CD68 (mon)	Neut	IgG	IgM	IgE	C3	C4d	C5b-9	CD41	Fibrin
**Biopsies at 2 h**																
**Allo Liver Tx**	B16407	B3408	2 h	**−**	**−**	**−**	**+**	**−**	**−**	**−**	**−**	**−**	**−**	**−**	**−**	**−**
**WT**	P3008	B16907	2 h	**++**	**+**	**++**	**−**	**++**	**+**	**+**	**−**	**++**	**−**	**+**	**+**	**+**
**GTKO/CD46**	P21708	B7708	2 h	**−**	**−**	**+**	**−**	**−**	**−**	**+**	**−**	**−**	**−**	**−**	**+**	**+**
	P22108	B7808	2 h	**−**	**−**	**++**	**−**	**+**	**+**	**+**	**−**	**−**	**−**	**++**	**−**	**++**
	P4009	B18908	2 h	**+**	**−**	**++**	**−**	**+++**	**−**	**++**	**−**	**−**	**−**	**−**	**+**	**++**
**Biopsies at necropsy**																
**Allo Liver Tx**	B16407	B3408	31 d	**−**	**+**	**−**	**+**	**++**	**−**	**−**	**−**	**−**	**−**	**−**	**−**	**−**
**GTKO/CD46**	P21708	B7708	7 d	**−**	**−**	**+**	**−**	**+++**	**−**	**+**	**−**	**−**	**−**	**−**	**+**	**++**
	P22108	B7808	6 d	**+**	**−**	**+**	**−**	**+++**	**+**	**+**	**−**	**−**	**−**	**−**	**+**	**+++**
	P4009	B18908	6 d	**−**	**−**	**++**	**−**	**+++**	**−**	**+**	**−**	**−**	**−**	**−**	**+**	**++**
**GTKO/CD46+CVF**	P3909	B18508	5 d	**−**	**−**	**++**	**−**	**+++**	**−**	**+**	**−**	**−**	**−**	**−**	**−**	**−**
**GTKO/CD46+CL**	P26708	B7908	<1 d	**−**	**−**	**+**	**−**	**++**	**−**	**−**	**−**	**−**	**−**	**−**	**++**	**+++**
	P26608	B8108	1 d	**+**	**−**	**+**	**−**	**++**	**−**	**+**	**−**	**−**	**−**	**+**	**−**	**+**

P = pig, B = baboon, Bx = biopsy, Neut = neutrophil, CL = clodronate liposomes, CVF = cobra venom factor, mon = monkey.

(−) = none; (+) = mild; (++) = moderate; (+++) = severe.

**Table 3:** Primary and secondary antibodies used for immunofluorescence staining of liver xenografts and native organs.

Name	Concentration	Source (Company)
**Primary antibodies**		
Rabbit anti-human CD3 (T-cell)	1∶100	Dako, Denmark
Mouse anti-human CD20 (B-cell)	1∶20	AbD Serotec, NC, USA
Mouse anti-pig CD68 (macrophage)	1∶25	AbD Serotec, NC, USA
Mouse anti-monkey CD68 (macrophage)	1∶100	Santa Cruz Biotech, CA, USA
Rabbit anti-human CD97 (neutrophil)	1∶100	Thermo Scientific, PA, USA
Goat anti-human IgG	1∶1000	K&P Lab, MD, USA
Goat anti-human IgM	1∶1000	K&P Lab, MD, USA
Mouse anti-human IgE	1∶100	Abcam, MA, USA
Mouse anti-human C3	1∶100	Santa Cruz Biotech, CA, USA
Rabbit anti-human C4d	1∶20	Abcam, MA, USA
Mouse anti-human C5b-9	1∶100	Abcam, MA, USA
Mouse anti-human CD41 (platelet)	1∶50	Abcam, MA, USA
Sheep anti-human fibrin	1∶100	Affinity Biol, Canada
**Secondary antibodies**		
Donkey anti-rabbit Alexa 488	1∶500	Molecular Probes, IL, USA
Donkey anti-mouse Cy3	1∶250	Jackson IR Lab, PA, USA
Donkey anti-goat Alexa 488	1∶500	Molecular Probes, IL, USA
Donkey anti-mouse Alexa 488	1∶500	Molecular Probes, IL, USA
Donkey anti-sheep Cy2	1∶100	Jackson IR Lab, PA, USA

Images were viewed at 20× magnification and captured using a Nikon confocal microscope (Nikon D-ECLIPSE C1, Tokyo, Japan). Metaphor Image Analysis software was used for image analysis [20]. At least 3 different areas from the same section were viewed and evaluated. An average of 12 different sections for each antibody stain was examined, and the positivity of staining was scored (Table 2). The percentages of positive staining area/total scanned area were calculated accordingly. Table 4 indicates the percentage of tissue stained by each antibody.

**Table 4:** Extent of immunofluorescence staining of liver xenografts and native organs.

Type of antibody	−	+	++	+++
Rabbit anti-human CD3 (T-cell)	<0.19%	0.2–1.3%	1.4–5.08%	
Mouse anti-human CD20 (B-cell)	<2.8%	2.9–4.6%		
Mouse anti-pig CD68 (macrophage)	<0.19%	0.2–0.9%	1–2.2%	
Mouse anti-monkey CD68 (macrophage)	<0.13%	0.14–0.24%		
Rabbit anti-human CD97 (neutrophil)	<0.07%	0.08–0.9%	1–1.49%	1.5–2.3%
Goat anti-human IgG	<0.29%	0.3–0.84%		
Goat anti-human IgM	<0.09%	0.1–7.9%	8–10%	
Mouse anti-human IgE	<0.01%			
Mouse anti-human C3	<0.03%	0.04–1.99%	2.0–5.01%	
Rabbit anti-human C4d	<0.01%			
Mouse anti-human C5b-9	<0.06%	0.07–0.29%	0.3–0.46%	
Mouse anti-human CD41 (platelet)	<0.08%	0.09–0.7%	0.8–1.0%	
Sheep anti-human fibrin	<0.9%	1–4%	5–14%	15–20.8%

Calculation of the percentages of tissue or cells stained was specifically and independently made for each antibody using Metaphor Image Analysis software.

### Electron microscopy

Liver tissue was fixed with 2.5% glutaraldehyde in PBS. Transmission electron microscopy was performed, as previously described [21]. Particular attention was paid to the examination of (i) the sinusoidal endothelial cells, Kupffer cells, and hepatocytes, (ii) the interaction between platelets and these cells, and (iii) fibrin deposition.

## Results

Donor livers were from pigs, except for one baboon alloTx. Thus, a brief description of normal pig liver histology is appropriate.

### Histological appearance of normal pig liver

**Figure 1:**
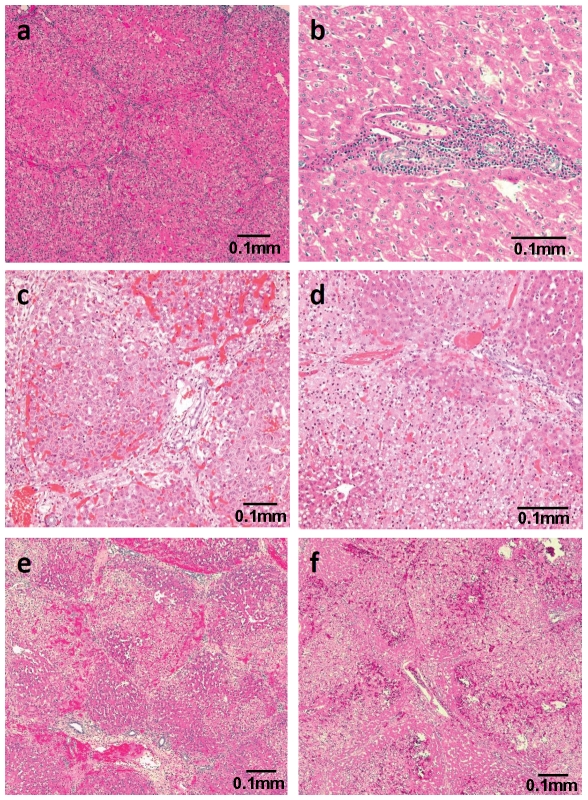
Histopathology (H&E) of (A) healthy (non-transplanted) pig liver, (B) baboon liver 31 days after allotransplantation, and (C–F) WT pig liver after pig-to-baboon xenotransplantation. A) Histomorphological appearance of a normal pig liver (×100). Distinct envelope of connective tissue surrounding hepatic lobules. B) Baboon-to-baboon allotransplantation (B3408) on post-operative day 31 (×200). Patchy, mild-to-moderate periportal lymphocytic infiltrates. C) WT pig-to-baboon liver xenoTx at 30 min (B16907) (×100). Significant hepatocellular vacuolar change, patchy sinusoidal congestion, and interlobular septal edema. D) WT pig-to-baboon liver xenoTx at 1 h (B16907) (×200). Severe hepatocellular vacuolar change, focal hepatocyte necrosis, and few thrombi. E) WT pig-to-baboon liver xenoTx at 3 h (B16907) (×100). Early coagulative necrosis, significant sinusoidal congestion, and large thrombus. F) WT pig-to-baboon liver xenoTx at 5 h (just before euthanasia) (B16907) (×100). Acute hemorrhagic coagulative necrosis, extensive hemorrhage, and large fibrin thrombi.

The hepatic lobules in the livers of pigs, unlike those of most other species, have a distinct, visually-recognizable envelope of connective tissue surrounding them, imparting a unique, individualized appearance to each structure (Figure 1A). In humans and NHPs, this prominent fibrous margination (which creates these histomorphologically-recognizable boundaries in swine) is absent; therefore, in these species, the identification of individual lobules as distinct structures is seldom possible.

### Histopathology of liver allografts (pig or baboon)

Recipients of two pig liver allografts (carried out to develop the surgical technique) did not receive any immunosuppression (Table 1), and were euthanized according to protocol on post-operative days 2 or 3. At necropsy, both livers showed minimal mononuclear portal and perivenular inflammation, and mild centrilobular congestion, suggesting early acute cellular rejection (not shown).

One immunosuppressed baboon with a liver allograft (Table 1) survived 31 days and was euthanized according to protocol. Biopsies were obtained at 2 h post-perfusion, on day 22, and at euthanasia (Table 1). The 2 h post-transplant wedge biopsy showed mild patchy areas of acute sinusoidal congestion/hemorrhage, possibly related to the biopsy procedure itself (not shown). Patchy, hepatocellular vacuolar change without evidence of an overt zonal patterning was also noted, possibly a consequence of organ reperfusion.

Tru-cut biopsy on post-operative day 22 showed unremarkable hepatocytes. One focal tiny nodular cluster of inflammatory cells was seen (not shown). There was a mild increase in the number of sinusoidal granulocytes present, especially in the frozen section. These may have represented passenger leukocytes as opposed to a mild response to hepatocellular insult, the former being suggested by correlation with clinical, hematologic, and clinical chemistry findings which were all normal [14].

At necropsy on post-operative day 31, the appearance of hepatocytes was unremarkable (Figure 1B). There were rare patchy mild-to-moderate periportal lymphocytic infiltrates. There was a mild CD20^+^ infilitrate (Table 2). The correlation with normal clinical, hematologic, and chemistry findings suggested that early acute rejection was unlikely, and an ascending pericholangitis might be a more likely diagnosis. No evidence of bile stasis or thrombosis was seen.

### Histopathology of liver xenografts

#### WT pig-to-baboon liver Tx without immunosuppressive therapy

Serial wedge biopsies were performed after WT pig-to-baboon liver xenoTx (Table 1).

- At 30 min after reperfusion, significant hepatocellular vacuolar change consisting primarily of large, clear, generally solitary vacuoles often displacing nuclei eccentrically, was noted (Figure 1C). There was marked congestion in many of the larger interlobular vessels, and a lesser degree of mild patchy sinusoidal congestion. A mild degree of increased interlobular septal edema was noted, which prominently accentuated the lobulated appearance of the organ. Infrequent small aggregates of polymorphonuclear cells were scattered throughout the sinusoidal spaces.
- At 1 h, severe hepatocellular vacuolar change was present, similar to that seen in the 30 min biopsy, although more prominent and extensive, with a centrilobular distribution (Figure 1D). Infrequent focal hepatocyte necrosis was seen. Scattered increased numbers of neutrophils were present within the sinusoidal spaces without aggregation, as noted at 30 min. A very small number of thrombi were observed.
- At 3 h, a distinct progression from vacuolation to early coagulative necrosis of the hepatocellular cytoplasm was observed (Figure 1E). This was characterized primarily by generally pale, more lightly eosinophilic staining. Scattered neutrophilic inflammatory cells remained present in these areas. Increased areas of sinusoidal congestion, extending in some regions to mild hemorrhage, were noted. A focal large thrombus was noted in a portal vessel.
- At 5 h, dark patchy areas were observed macroscopically. Therefore, normal (light) and abnormal (dark) areas were biopsied separately. In the *light* area biopsies, multiple regions located primarily in subcapsular areas were noted in which focal acute hemorrhagic coagulative necrosis was present, sometimes with a centrilobular distribution (not shown). In other more central lobules, there was more subtle evidence of hepatocellular vacuolar change with mild associated eosinophilic cytoplasmic pallor. One focus of subcapsular hemorrhagic necrosis had a distinct wedge-shaped appearance, suggesting possible infarction. In the *dark* areas, although some relatively unaffected portions of hepatic parenchyma were noted, there was a focally extensive region of significant hepatocellular degeneration characterized by a combination of severe vacuolar change and frank coagulative necrosis with extensive but patchy hemorrhage (Figure 1F). As with other sections, the same zonal patterning was noted, with periportal hepatic zones appearing spared of insult. A large early fibrin thrombus was noted in a portal vessel.

#### GTKO or GTKO/CD46 pig-to-baboon liver Tx with immunosuppressive therapy

Six longer-surviving (>1 day) recipient baboon liver biopsies (Tables 1 and 5) were examined at 2 h and at necropsy (4–7 days).

**Table 5:** Macroscopic observations at necropsy of hemorrhage in native organs and body cavities after genetically-engineered pig liver xenotransplantation in longest-surviving baboon recipients.

Baboon #	Survival (days)	Blood-stained fluid in the chest	Heart	Lung	Blood-stained fluid in the abdomen	Melena stools	Small intestine	Kidney	Lymph nodes (mesenteric)
B3108	6	+/−	−	−	+++	+	++	−	−
B3208	4	++	−	+++	++	−	+	−	−
B7708	7	++	−	+++	++	−	−	−	−
B7808	6	+/−	−	−	++	+	++	−	−
B18508	5	+/−	−	++	+++	−	−	+/−	−
B18908	6	+	+	−	+++	+	+++	+	−

(−) = none; (+/−) = very mild; (+) = mild; (++) = moderate; (+++) = severe. All necropsies were performed by the same surgeon (B.E) and pathologist (E.K). The spleen was not studied since splenectomy was performed in all recipients immediately after reperfusion of the liver graft, except in B3108.

**Figure 2:**
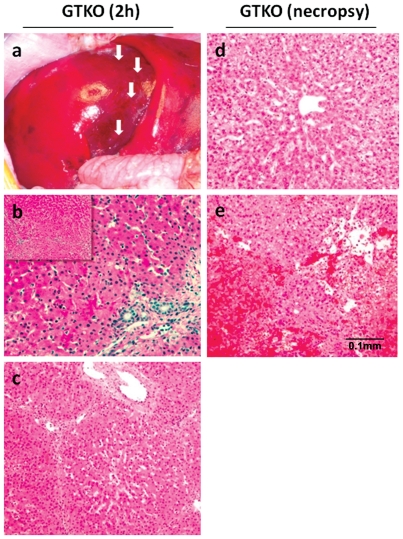
Macroscopic and microscopic appearance of GTKO and GTKO/CD46 pig livers 2 hours after perfusion of the graft and at necropsy. A) B18508 (2-hour post-reperfusion). Macroscopic appearance of well-perfused pig liver. Lobular dark areas were noted in the middle of the liver (arrows) suggesting an arterio-embolic event. B) B18908 (2-hour post-reperfusion). Prominent increase in neutrophils (H&E ×200). Small panel at top left (H&E ×100). C) B18508 (2-hour post-reperfusion). Intra-hepatocellular vacuolar change. The pig liver in B18508 demonstrated the most severe degree of involvement (although the change was still relatively mild and subtle in extent) (H&E ×200). D) B18908 (light area at necropsy). Vacuolar hepatocellular cytoplasmic change (H&E ×200). E) B18508 (dark area at necropsy). Extensive hemorrhage and hemorrhagic necrosis, sparing only focal groups of hepatocytes in the portal regions (H&E ×200). Solid black bar indicates 0.1 mm for figures 2B, 2C, 2E, and 2F. Macroscopic appearance of livers at necropsy (day 7), with dark patchy areas and a yellowish color suggesting cholestasis in some cases, has been already shown [14].

At 2 h, all of the livers were well-perfused, although in one case (B18508) there was a macroscopic lobular darker area noted that could represent a possible embolic event during reperfusion (Figure 2A). Histomorphological changes at 2 h were minimal or mild. Differential sinusoidal dilatation was present in some cases, but generally did not differ from pre-Tx control biopsies. There was moderate variation in the number of neutrophils and neutrophilic clusters seen within the sinusoids, which was most prominent in B18908 (Figure 2B). In some cases this did not differ significantly from pre-Tx biopsies. In a similar fashion, some livers demonstrated what appeared to be patchy mild hepatocellular and canalicular bile stasis, but the pre-Tx ‘controls’ also showed similar foci, suggesting this was not an immediate experimental consequence. Of most apparent significance was the presence of intra-hepatocellular vacuolar change which was more extensive in the 2 h biopsies than in the pre-Tx samples (generally absent from the latter) (Figure 2C), and was thought likely to be due to ischemia/reperfusion injury. Based on the structural appearance of nuclei and the generally normal tinctorial nature of hepatocyte cytoplasm, there was no indication of frank hepatocellular necrosis.

At necropsy, the macroscopic appearance of livers was generally normal or yellowish, indicating cholestasis (*light* areas), with patchy dark areas. Therefore, individual side-by-side comparisons of *light* and *dark* areas were made. The synopsis below summarizes the changes and variations seen in each area, and also provides an overall comparison between the two.

Light areas: Although large regions of these sections were normal, occasional coalescent areas demonstrated distinct, multifocal areas of coagulative necrosis. Often admixed with this change were varying degrees of hemorrhage, though the extent and severity of such lesions varied significantly between livers. Frequently, a distinctive central lobular pattern was noted, although in some cases apparent foci of individual hepatocellular degeneration were also observed. Vacuolar hepatocellular cytoplasmic change (and in some cases distention) of unknown etiology was also present in several cases, being most prominent in B18908 (Figure 2D). Small fibrin thrombi were seen not infrequently in portal vessels, although typically not extensive in their distribution. Also present and variable in extent (although generally mild) were features of ductal and occasional canalicular bile stasis.

Dark areas: The microscopic changes seen in the *dark* regions were generally similar to those in light areas, but with much more severe/extensive necrosis and evidence of vastly increased hemorrhage. Although variable in degree (as with *light* region biopsies), total lobular hemorrhagic necrosis was often seen or there was sparing only of focal groups of hepatocytes in the portal regions (Figure 2E). Again, thrombi were occasionally noted, but were not extensive. In areas in which there was not massive lobular hemorrhagic necrosis, predilection for central involvement of the lobule was again seen. In some cases in which viable parenchyma remained, hepatocellular cytoplasmic vacuolar change and distention (as seen in *light* areas) was seen, most prominently in B18908. In the pig liver in B18508, morphological change consistent with a distinct area of hepatic infarction was present (Figure 2A). This was characterized by large multifocal fibrin thrombi and severe hepatocyte disassociation/individualization. In regions of this section (often underlying the hepatic capsule), numerous punctate structures consistent with bacterial colonies were observed, presumed to be associated with pre-mortem retrograde migration of enteric bacterial flora through thrombosed vessels. No inflammation was noted in association with the presence of these organisms.

### Immunohistopathology of liver xenografts

The liver grafts were examined at 2 h and at necropsy (Table 2).

#### Cellular infiltrates

**Figure 3:**
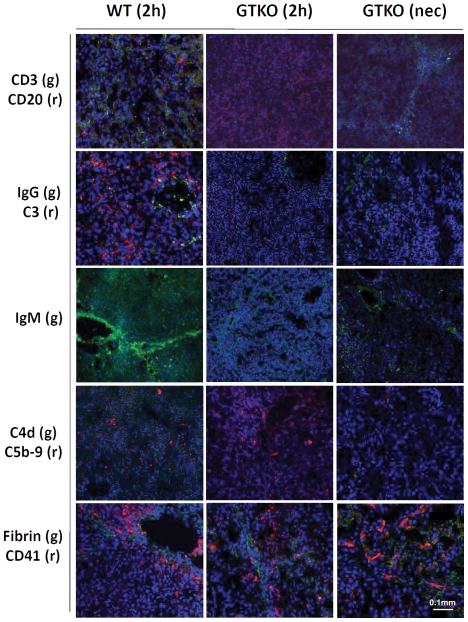
Immunohistological assessment of liver xenografts. Cellular infiltrates (CD3, CD20), antibody (IgG and IgM) and complement (C3, C4d, C5b-9) deposition were obvious in the WT pig liver at 2 hours, but were minimal or absent in GTKO and GTKO/CD46 pig livers. Fibrin and platelets were present in WT or GTKO and GTKO/CD46 pig livers regardless of post-Tx time, immunosuppression, and genetic modification of the organ-source pig. For details, see Table 2. (g) = green, (r) = red. Solid white bar indicates 0.1 mm for all figures.

When HAR occurred in the WT pig liver xenograft, there was a significant cellular response, as well as antibody and complement deposition (Table 2 and Figure 3).

In the GTKO/CD46 pig liver xenografts at 2 h, there was no T or B cell infiltrate except a very mild CD3^+^ infiltrate in one case (B18908) (which correlated with light microscopy findings in pre-Tx and 2 h biopsies with similar cellular infiltration and neutrophil clusters [see above]). This liver showed the most prominent neutrophil-staining among the 2 h biopsies (Table 2). At necropsy, there was a mild increase in CD3^+^ staining in one case (B7808). Between the 2 h and necropsy biopsies, we observed a significant increase in neutrophil-staining (even in the liver allograft recipient which was negative pre-Tx) (Table 2).

To decrease liver macrophages (Kupffer cells), in 2 cases (B7908 and B8108) clodronate liposomes were administered to the organ-source pig 24 h before excision and Tx of the liver. The combination of clodronate liposomes followed by ischemia-reperfusion appeared to be toxic to the liver since primary non-function occurred in both cases [14]. Immunohistological staining confirmed that macrophage depletion was not completely successful (Table 2), though there were fewer macrophages in the 2 h biopsies than in other pig livers.

#### Antibody and complement deposition

The WT pig liver showed deposition of IgG, IgM, C3, and C5b-9 (Table 2 and Figure 3). There was no IgG deposition in GTKO/CD46 pig livers at 2 h and at necropsy, except in one case (B7808) which showed minimal deposition (+ = <1%, Tables 2 and 4). IgM deposition was observed in almost all liver xenografts at 2 h and at necropsy, although deposition was minimal in many cases (+ = 1–8%, Table 4); in one case (B18908), it reached up to 10% (++) on the 2 h biopsy (Figure 3 , Tables 2 and 4). There was no correlation between euthanasia time-points and IgM deposition. In an effort to understand whether the immediate thrombocytopenia seen after reperfusion was related to mast or eosinophil cell activation, liver tissues were stained with an IgE antibody; all 2 h and necropsy biopsies stained negative (Table 2). Both 2 h and necropsy samples from GTKO/CD46 pig livers were negative for complement deposition (C3, C4d, and C5b-9), except in B7808 at 2 h and B8108 at 1 day (Table 2 and Figure 3), where deposition was minimal (<1%) (Table 4).

#### Thrombotic microangiopathy

To assess the extent of thrombotic microangiopathy (platelet aggregation and fibrin deposition) in the grafts, the grafts were stained for CD41 (platelets) and fibrin. All, including the WT graft, stained positive for platelets and fibrin (Table 2) even in the 2 h biopsies. (The 2 h and necropsy biopsies of the baboon allograft (B3408) showed no platelet or fibrin staining, suggesting that there was neither platelet aggregation nor fibrin deposition after alloTx) (Table 2). In some pig liver xenografts (B7808 and B7908), fibrin deposition reached up to 20% (+++) at necropsy (Tables 2 and 4 and Figure 3). However, no platelet or fibrin staining was seen in the pig liver in B18508, which had been treated with cobra venom factor (Table 2), although this baboon showed the same degree of thrombocytopenia as the other recipients [14].

### Ultrastructure of liver xenografts

**Figure 4:**
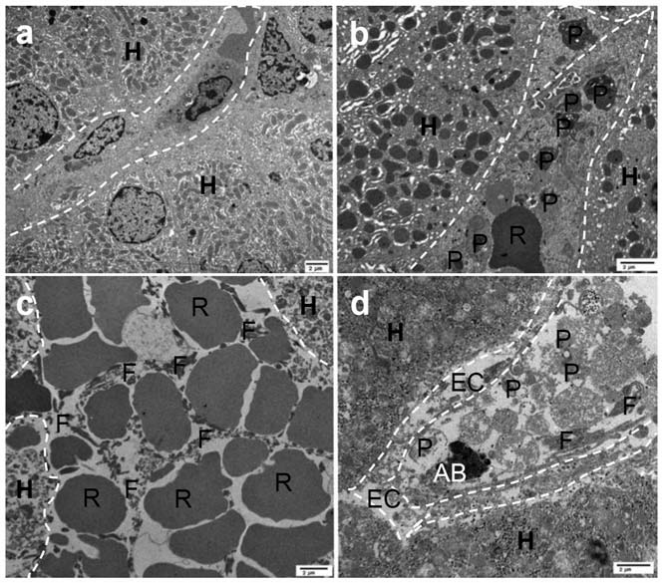
Electron microscopic appearance of liver xenografts. A) Ultrastructure of GTKO/CD46 liver before excision from the pig. B) (2 h post-reperfusion). Normal appearance of hepatocytes (H) with widespread platelet (P) aggregation and deposition along liver sinusoidal endothelial cells. C) (necropsy, dark area). Red blood cells (R) with fibrin deposition (F) indicating fibrin clots in the sinusoids. D) (necropsy, light area). Platelet, platelet/monocyte and platelet/necrotic cell aggregates along liver sinusoidal endothelial cells, and patchy endothelial cell death. Dashed lines indicate endothelial cells lining the sinusoids. AB = apoptotic body, EC = endothelial cells, F = fibrin, H = hepatocytes, P = platelets, R = red blood cells. The line at the bottom right indicates 2 µm.

Electron microscopy was not carried out in the WT liver that underwent HAR. With GTKO/CD46 pig livers, comparisons were made between pre-perfusion (pre-Tx), 2 h post-perfusion, and necropsy (light and dark area) biopsies (Figure 4). At 2 h, hepatocytes and sinusoidal endothelial cells were relatively normal (Figure 4B). However, deposition of platelets was observed along the sinusoid walls. At necropsy, a significant presence of red blood cells and fibrin was observed in dark areas, indicating large fibrin clots (Figure 4C), whereas light areas showed deposition of platelets along the sinusoidal endothelial cells together with mononuclear cells and other unidentified necrotic cells (Figure 4D). In the light areas, patchy endothelial cell death was documented. We did not observe any platelet phagocytosis by hepatocytes or liver endothelial cells, as previously shown by Burlak et al. [22] and Peng et al. [23] in their *in vitro* models. However, our biopsies were limited and randomized, which could explain the lack of this finding.

### Histopathology of the native organs in liver xenograft recipients

Hemorrhage was sometimes clinically manifest by petechiae in the skin and buccal mucosa, and by melena stools [14]. Native organs of 6 longer-surviving (>1 day) recipients were macroscopically assessed and biopsied (wedge) under direct vision at necropsy (Tables 1 and 5), and were examined by light microscopy for morphological changes. In particular, macroscopic evaluation of hemorrhage in body cavities and native organs was carried out (Table 5). In 3 of the 6 recipients, the native organs were stained for platelet aggregation and fibrin deposition (Table 6).

**Table 6:** Immunohistopathological features of thrombotic microangiopathy in recipient native organs at necropsy.

Baboon#	Time of Bx	CD41 (Platelets)	Fibrin
**Heart**			
B7708	day 7	−	−
B7808	day 6	−	−
B18908	day 6	++	−
**Lungs**			
B7708	day 7	++	−
B7808	day 6	++	−
B18908	day 6	++	−
**Small intestine**			
B7708	day 7	−	−
B7808	day 6	−	−
B18908	day 6	+	−
**Kidneys**			
B7708	day 7	−	−
B7808	day 6	−	−
B18908	day 6	++	+
**Lymph nodes (mesenteric)**			
B7708	day 7	−	−
B7808	day 6	−	−
B18908	day 6	+	−

B = baboon, Bx = biopsy, n.a = not available.

(−) = none; (+) = mild; (++) = moderate; (+++) = severe.

Clear signs of hemorrhage were observed in several sites in every baboon, presumably a direct result of severe thrombocytopenia. Blood-stained fluid was present in the thoracic cavity (from very mild-to-moderate) and in the abdominal cavity (moderate-to-severe) (Table 5 ). In order to identify whether there was a major bleeding site intraperitoneally, we performed relaparotomy in one case on post-operative day 4 (B18508). We observed that there was no major bleeding site, and all anastomoses were intact, but blood was oozing from the peritoneum and other soft tissues. All anastomoses remained intact in all baboons at necropsy.

#### Heart

**Figure 5:**
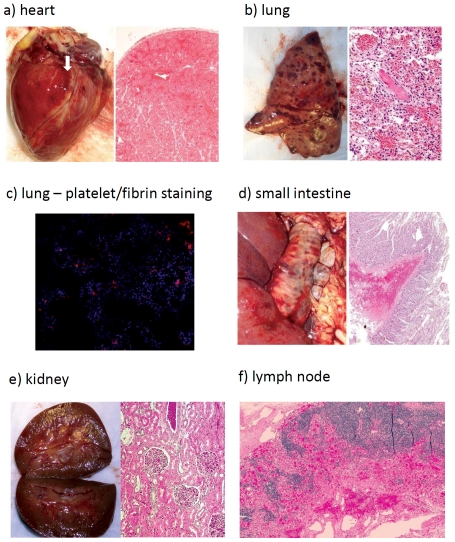
Macroscopic and microscopic appearances of native organs. A) Heart: Left panel, macroscopic appearance of heart (B18908). Arrow indicates macroscopic bleeding in the myocardium (B18908). Right panel, subendocardial hemorrhage within the myocardium as well as on the epicardial surface (H&E ×100). B) Lungs: Left panel, macroscopic appearance of lung in which there was patchy bleeding (B3208) (Table 5). Right panel, acute congestion, focal atelactasis, multiple thrombi with no features of inflammation (H&E ×200). C) Staining for platelets (CD41^+^) was positive in lungs, suggesting platelet aggregation and migration. This phenomenon was observed in lungs regardless of bleeding, but was present in other native organs only when bleeding had occurred (Table 6) (×100). D) Small intestine: Left panel, macroscopic hemorrhage in the wall of small intestine. Right panel, although the mucosa appeared normal, prominent submucosal hemorrhage was noted (B18908) (H&E ×100). E) Kidneys: Left panel, kidneys were macroscopically normal, except in one case (B18908) which showed small, patchy petechiae. Right panel, acute glomerular congestion associated with tubular and interstitial hemorrhage (H&E ×100). F) Lymph nodes: Although the macroscopic appearance was normal, lymph nodes in one case (B18508) showed hemorrhage in the hilar and medullary regions (H&E ×200).

Macroscopically, there was no hemorrhage observed in the myocardium, except in B18908 (Table 5 and Figure 5A). Microscopically, sections of heart examined were essentially normal with the exception of two (see below). There were no features suggesting inflammation, myofiber degeneration, or fibrin thrombi.

Microscopically, B3108 showed several patchy areas of minimal-to-mild congestion and hemorrhage. Consistent with the macroscopic necropsy appearance of the heart, B18908 had extensive hemorrhage throughout the organ (Figure 5A). This was most prominent in one large subendocardial area, although patchy foci of hemorrhage were also noted within the myocardium as well as on the epicardial surface. In numerous areas the hemorrhage and related edema caused a mild separation of individual myofibers by expanding the interstitial space between them. No platelet aggregation or fibrin deposition was seen, except in B18908, where there had been extensive hemorrhage (Table 6).

The hemorrhage seen in these two cases (mild or severe) was likely spontaneous as a result of severe thrombocytopenia in the baboon, and therefore a secondary manifestation of a systemic problem (rather than representing primary cardiac disease).

#### Lungs

Macroscopically, hemorrhage in the lungs was patchy, but moderate-to-severe (Table 5 , and Figure 5B). Microscopy showed patchy mild acute congestion, occasional focally-extensive atelactasis (B3108), multiple (numerous) thrombi in small interstitial vessels (most dense frequency in B7808), with no features of inflammation. No morphological evidence of platelets was noted under light microscopy, but immunohistological staining was always significantly positive (Table 6) (Figure 5C). In one case (B3208), there was focally-extensive hemorrhage (the most severely hemorrhagic lung of all baboons) (Figure 5B); scattered alveolar macrophages engulfing granular material were also observed, which could indicate platelets.

#### Small intestine

Macroscopically, hemorrhage was extensive in the walls of the small intestine (Table 5), and 3 of 6 recipients had melena stools. Microscopically, there was moderately extensive submucosal (B7708, B18908) and mild patchy serosal hemorrhage and congestion with no evidence of any mucosal involvement, even in the presence of melena stools. By causing dissection, submucosal hemorrhage distended the submucosal space (especially in B18908) (Figure 5D). There were no thrombi, and the mucosa looked normal. Immunofluorescence staining was negative, except for platelets in B18908, which had the most prominent hemorrhage (Table 5).

#### Kidneys

Macroscopically, the kidneys showed no signs of hemorrhage, except in one case (B18908) (Table 5). On light microscopy, there was mild capillary congestion, mild tubular proteinosis, and, in one case, infrequent small fibrin-microthrombi (B3108) (both interstitial and glomerular vessels). The patchy mild-to-moderate tubular proteinosis could have been an effect of the infusion of human albumin or of protein loss (B7808 and B18908). There were no fibrin thrombi in any case. In the only kidney that showed hemorrhage (B18908), acute severe glomerular capillary congestion associated with tubular and interstitial hemorrhage was present (Table 5 and Figure 5E). Granular and hyaline-appearing glomerular proteinaceous casts possibly suggested moderate hemoglobinuric nephrosis. This kidney was positive for platelet and fibrin staining (Table 6).

#### Lymph nodes

Mesenteric lymph nodes were collected during necropsy. Although macroscopic evaluation was not easy, no obvious hemorrhage was noted (Table 5). Microscopically, patchy mild cortical hemorrhage and congestion was present in B3108, and significant congestion and bleeding in the hilum and deeper medullary region was present in B18508. In other cases, no features of hemorrhage were seen. No thrombi were observed in any cases (Figure 5F).

## Discussion

The main aim in the pig allotransplants was to study pig liver anatomy and investigate the surgical technique, and therefore no immunosuppressive therapy was administered. As expected, early features of acute cellular rejection were observed on days 2 and 3. The baboon allotransplant was carried out (i) to study baboon liver anatomy and surgical technique, and (ii) to test our clinically-applicable immunosuppressive regimen (Table 1). No significant features of humoral or cellular rejection were seen.

The one WT pig-to-baboon liver xenoTx was carried out to study the development of HAR. Cellular infiltration and antibody and complement deposition were confirmed (Table 2 and Figure 3), as previously reported by Luo et al. [19]. In their experience, more rapid vascular rejection was seen in the WT pig-to-baboon model than in the WT pig-to-rhesus monkey model. However, heterotopic Tx was performed in the former model, but orthotopic Tx in the latter. The removal of the native liver almost certainly reduced the amount of native complement (90% of which is produced by the liver) that could participate in the rejection process; Hara et al have demonstrated that pig complement has a significantly reduced effect on a pig xenograft than human complement [24].

There have been no previous studies in which sequential liver biopsies have been taken during the development of HAR. Therefore, the present study describes, for the first time, the evolution of HAR. Ramirez et al. [12], [13] observed severe edema and sinusoidal congestion in 3 WT pig liver transplants in baboons, with destruction of vascular and sinusoidal endothelium and parenchymal hemorrhage. Their immunohistological findings were similar to those in the present study [12].

Biopsies obtained from GTKO or GTKO/CD46 pig livers did not show signs of HAR in the 2 h biopsies. At this time-point, light microscopic findings were generally normal and there was no cellular infiltrate or antibody and complement deposition, except minimal IgM deposition (Tables 2 and 4). Variable extents of sinusoidal dilatation and hepatocellular vacuolation were seen, but were not severe. Electron microscopy confirmed almost normal hepatocytes and endothelial cells.

There is scarce experience of GE pig liver xenoTx in the literature [11]. Ramirez et al, using human CD55 or CD55/CD59/H-transferase-expressing pigs [12], [13], demonstrated the absence of HAR in 2 h liver biopsies. However, although they reported no deposition of C3 and C5b-9, there was positivity for immunoglobulin and C4 deposition without any information regarding the isotype of the immunoglobulin or extent of deposition [12].

In the present study, there were significant changes in the livers at necropsy, but these were largely related to hemorrhage within the liver, rather than a result of an immune response, i.e., antibody-mediated or cellular rejection. Some lesions may have been associated with hepatocellular ischemia due to anemia and organ hypoperfusion. The hemorrhage noted was most likely directly associated with the profound thrombocytopenia that developed rapidly in all baboons, though a role for immune-mediated injury cannot be totally excluded. Except for minimal IgM deposition, however, there was no or minimal cellular infiltration or antibody and/or complement deposition (Tables 2 and 4 and Figure 3). Nevertheless, there is a possibility that patches of hemorrhagic necrosis may have masked features of immune injury.

**Figure 6:**
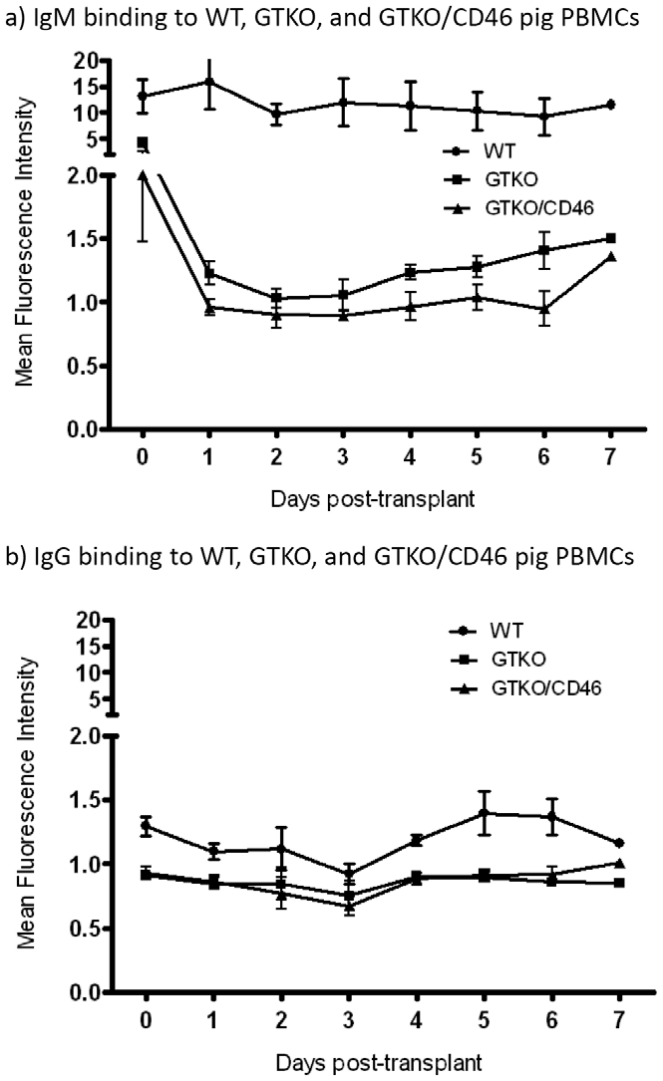
Average anti-nonGal antibody levels (IgM and IgG) in 4 baboons. Serum from the longest-surviving recipient baboons (n = 4; B7708, B7808, B18508, and B18908) was drawn daily (pre-Tx to postoperative day 7), and stored at −80°C until processed. Binding of (i) anti-nonGal IgM and IgG antibodies to GTKO and GTKO/CD46, and of (ii) anti-pig antibodies (Gal and nonGal), to WT pig peripheral blood mononuclear cells (PBMCs) was measured by flow cytometry [29]. A) IgM binding against WT, GTKO, and GTKO/CD46 pig PBMCs: Significantly increased binding to WT pig PBMCs. However, GTKO and GTKO/CD46 pig PBMCs showed decreased binding throughout the study. B) IgG binding against WT, GTKO, and GTKO/CD46 pig PBMCs: Although IgG binding against WT pig PBMCs was slightly increased; there was no significant difference between bindings against GTKO and GTKO/CD46 pig PBMCs.

The observation that anti-nonGal antibody levels did not increase (although this might not be expected within 4–7 days) lends support to this conclusion (Figure 6). *In vitro* antibody binding to porcine aortic endothelial cells from GTKO and GTKO/CD46 pigs was also measured [24], confirming minimal or absent antibody binding. This further finding suggests (though does not prove) that antibody-mediated injury did not cause the changes seen in the liver grafts.

Similar observations were made by Ramirez et al. [12]. In their 4-day survivor, there were no features of rejection, and liver lobular architecture was preserved, although hemorrhage was noted. Similar to our findings, in Ramirez et al's 8-day survivor, hemorrhage and hemorrhagic/ischemic necrosis in the liver was seen, but they emphasized the absence of features of rejection [12].

Observations in the present study with light and electron microscopy and with immunohistological studies with confocal microscopy indicated that there was deposition of fibrin and platelets in the liver grafts, which increased between the 2 h biopsies and necropsy (Tables 2 and 4 and Figure 3). Significant platelet aggregation was seen in the liver sinusoids. Electron microscopy confirmed fibrin deposition in the liver especially at necropsy in the dark areas. In the light areas, fibrin deposition was noted as well, but was significantly less than in the dark areas. Fibrin deposition was not seen particularly in the native organs, except in the kidneys of B18908 [Table 6 ]). Flow cytometry studies by Ezzelarab et al have demonstrated extensive platelet-WBC aggregation in the peripheral blood (Ezzelarab et al, submitted).

Peng et al. [23] recently showed *in vitro* that pig hepatocytes, liver sinusoidal and aortic endothelial cells from GTKO or WT pigs induced moderate aggregation of baboon platelets, which correlates with the findings of Burlak et al. [22]. Although recent *in vitro* studies have indicated (by electron and confocal microscopy) platelet phagocytosis by hepatocytes and liver sinusoidal endothelial cells [22], [23], we were unable to confirm this phenomenon in our ultrastructural studies. We suggest this may be a result of differences between *in vitro* and *in vivo* models. *In vitro* studies may provide better and more repeated access to biopsies for electron microscopy [22], and platelets can be more easily tagged to follow their destiny [22], [23].

Nevertheless, it is possibly surprising that platelets are phagocytosed by liver sinusoidal endothelial cells. Soluble components and colloidal particles are cleared from the blood by hepatic endothelial cells by receptor-mediated endocytosis [25], [26], but these particles are <0.23 µm in diameter (almost 10-fold less than the normal platelet diameter). Furthermore, in our *in vivo* studies, platelet-platelet and/or platelet-WBC aggregation was documented in the blood and in the graft, possibly making it more unlikely that platelets would be successfully phagocytosed. However, platelets may migrate through the fenestrations or between the sinusoidal endothelial cells, which could possibly enlarge under certain circumstances, and these cells may change their behavior, possibly as a result of activation, ischemia/reperfusion injury, inflammation, etc. How platelets become activated (possibly through a specific receptor, such as the Ashwell receptor [27] or through CD47-SIRPalpha interaction [28]) or aggregate on the sinusoidal endothelial cells remains uncertain and require investigation (Lin et al, submitted).

We also studied the native organs of the recipient baboons. Macroscopic and microscopic assessments strongly suggested bleeding had occurred as a result of the severe thrombocytopenia. The most affected organs were the lungs and small intestine (Table 5 , Figure 5), but petechiae of the skin, and melena stools were common. Immunohistological studies confirmed platelets in the lungs regardless of the presence of bleeding, and in other native organs in which bleeding had occurred (Figure 5B and Table 6).

In conclusion, after GTKO and GTKO/CD46 pig liver Tx in baboons, the rapid development of a profound thrombocytopenia was by far the major problem seen, and was suggested as the major causative factor in the hemorrhage that occurred, not only in the pig liver grafts, but also in several native organs and body cavities. The histopathologic features described can largely be explained on this basis. However, the factors influencing the activation and consumption of platelets, and therefore the development of coagulopathy, need to be studied.

Since the features of rejection were minimal or absent, we believe that, if platelet activation and aggregation/phagocytosis can be prevented, possibly by further genetic modification of the pigs or by novel therapeutic agents, bridging by a pig liver to alloTx may become a feasible clinical option.
